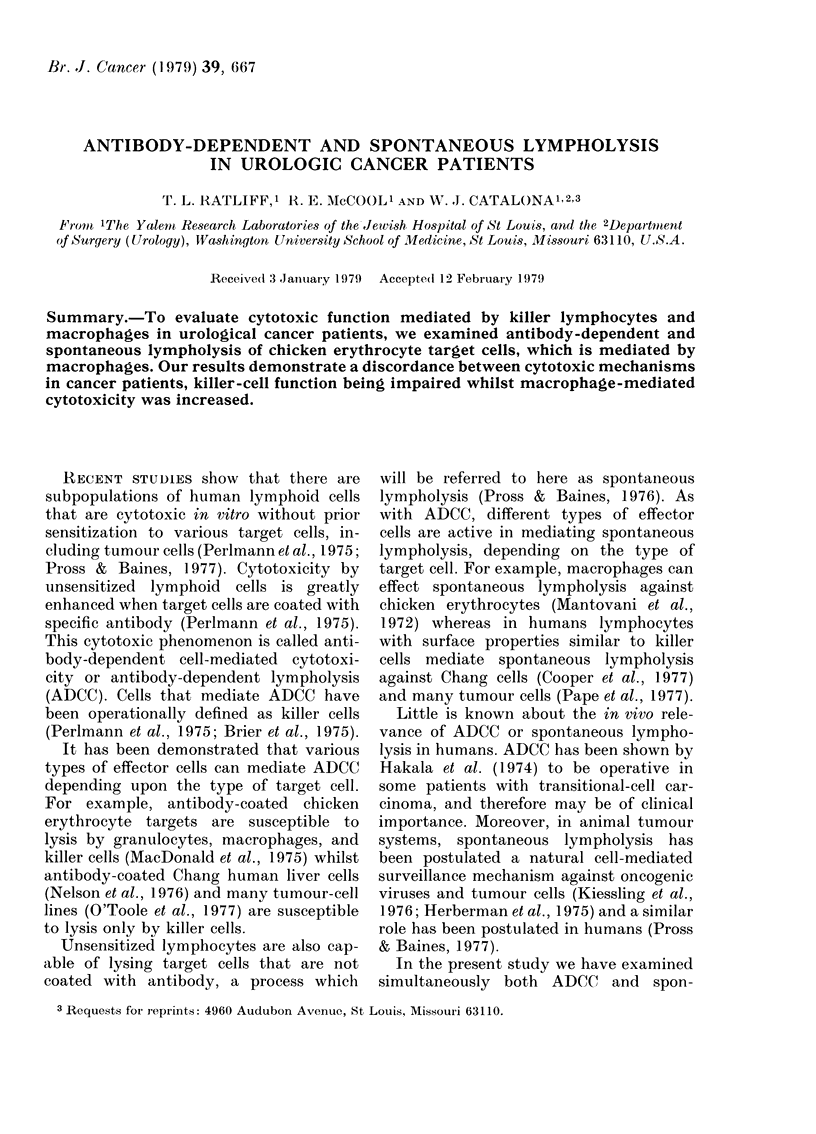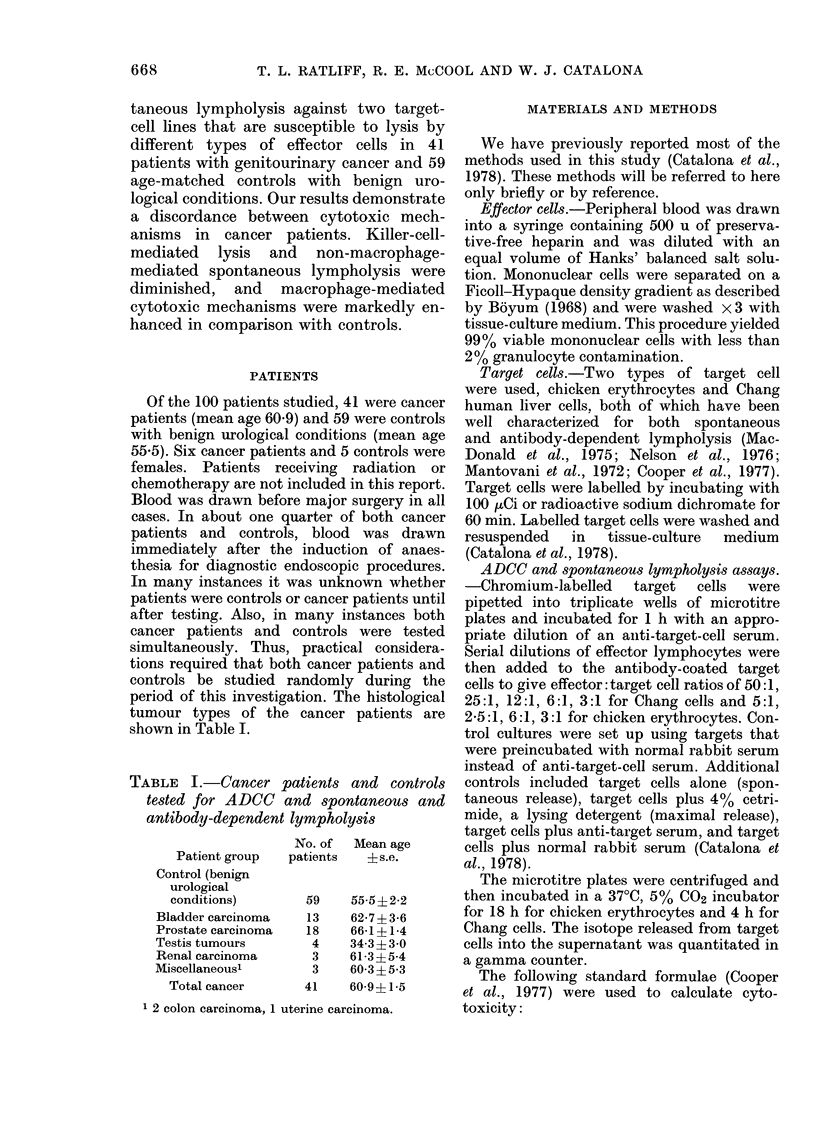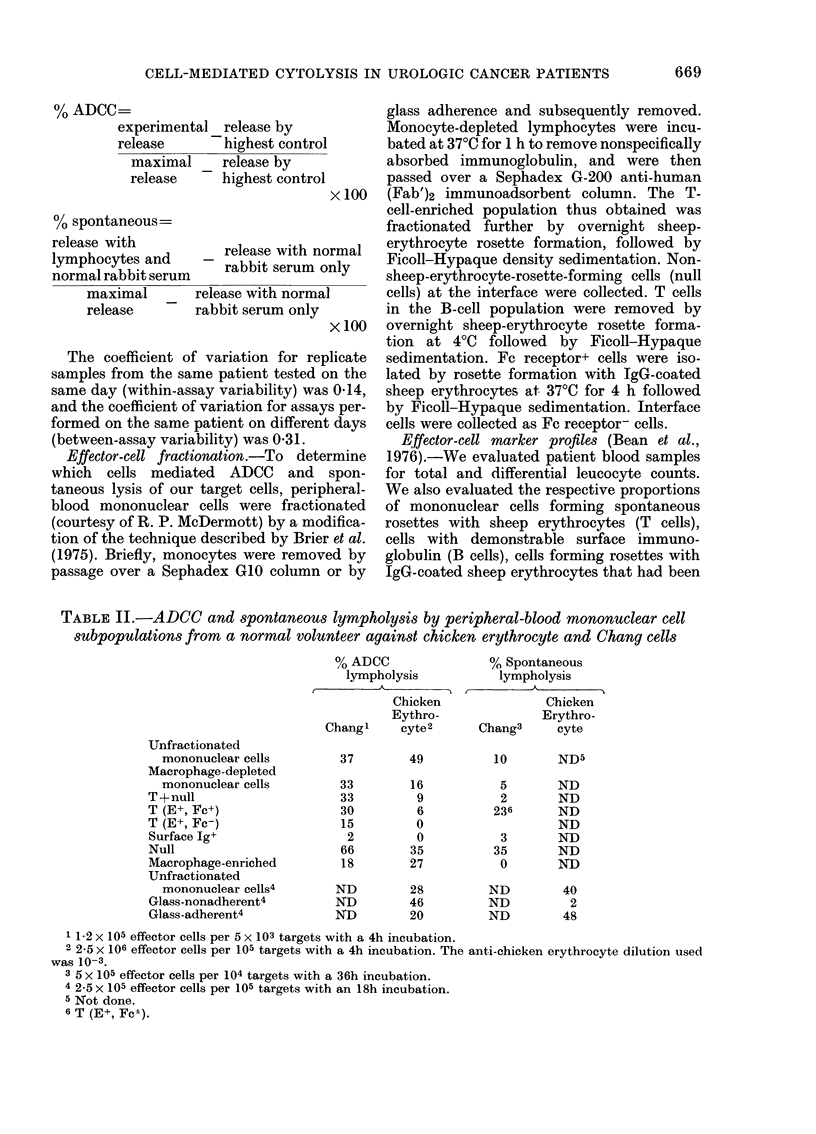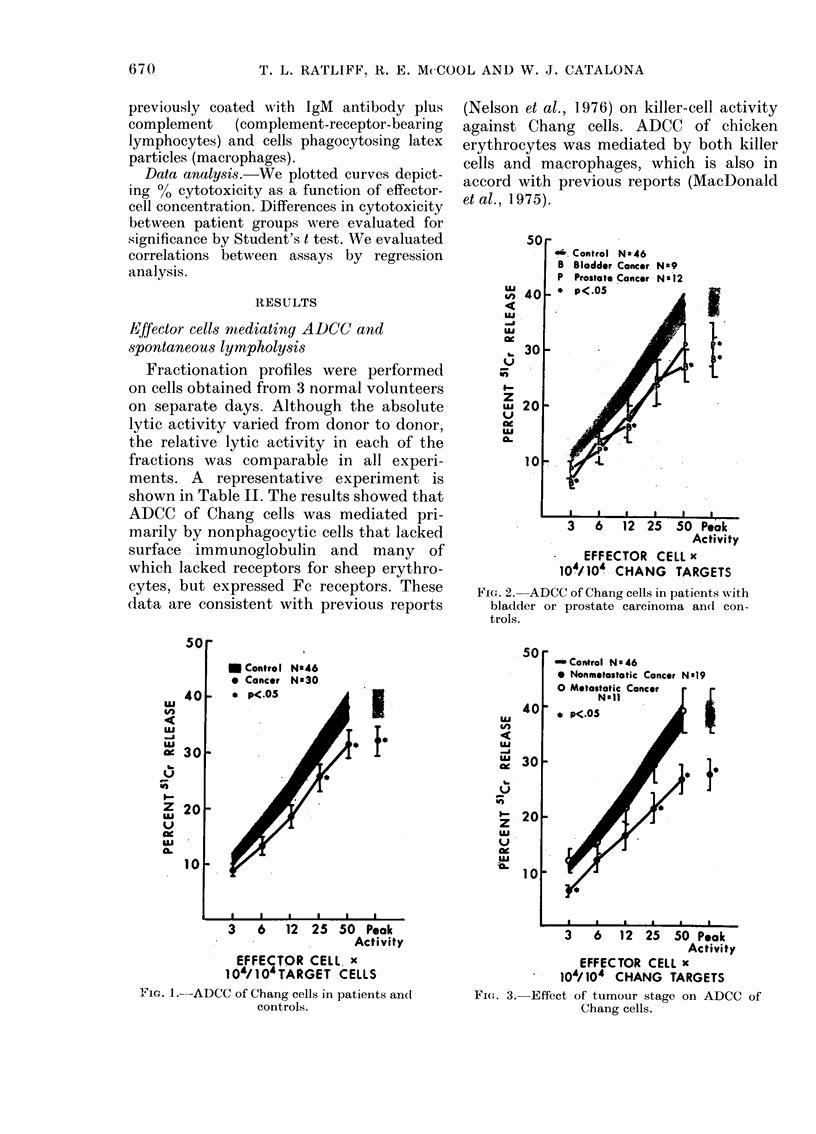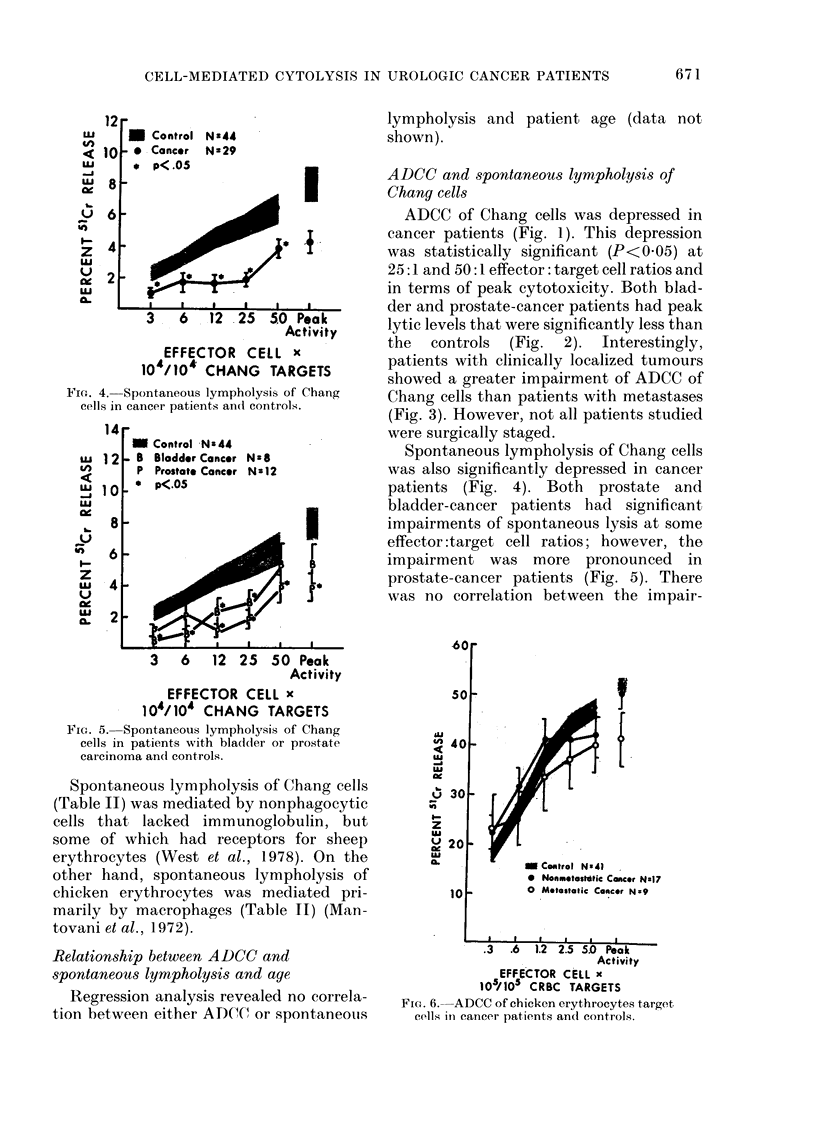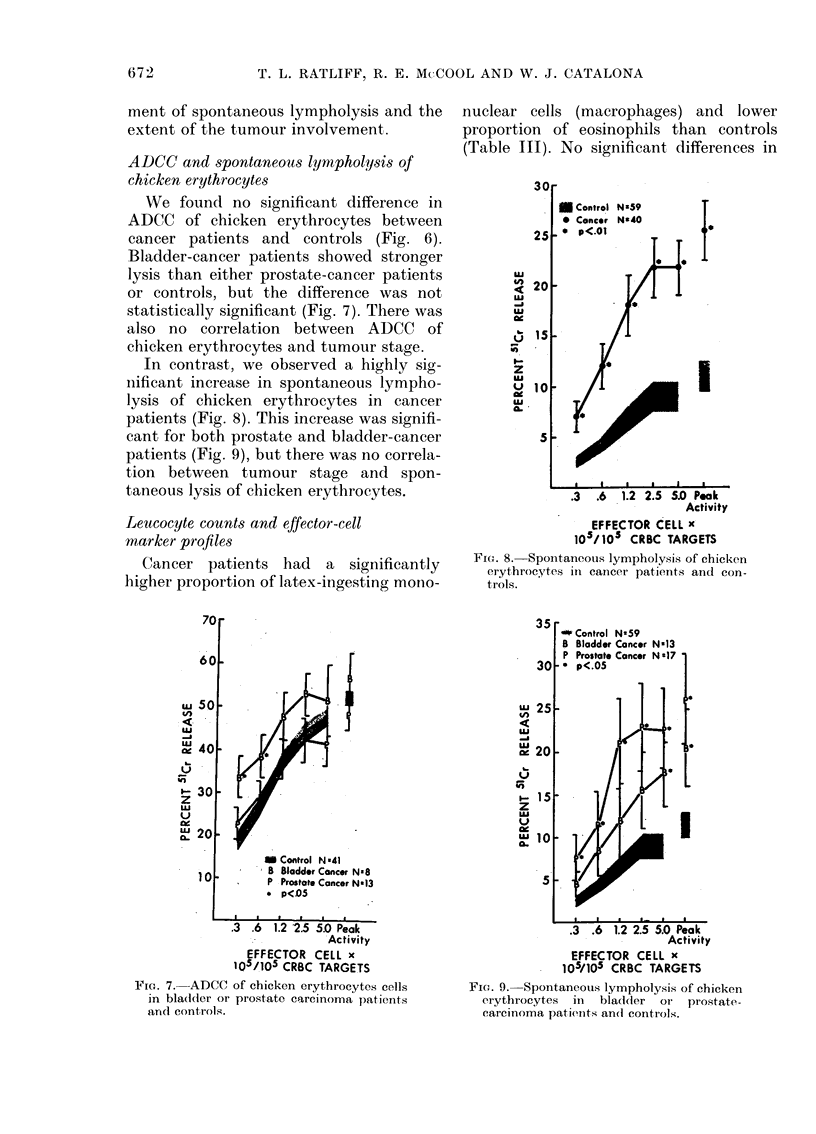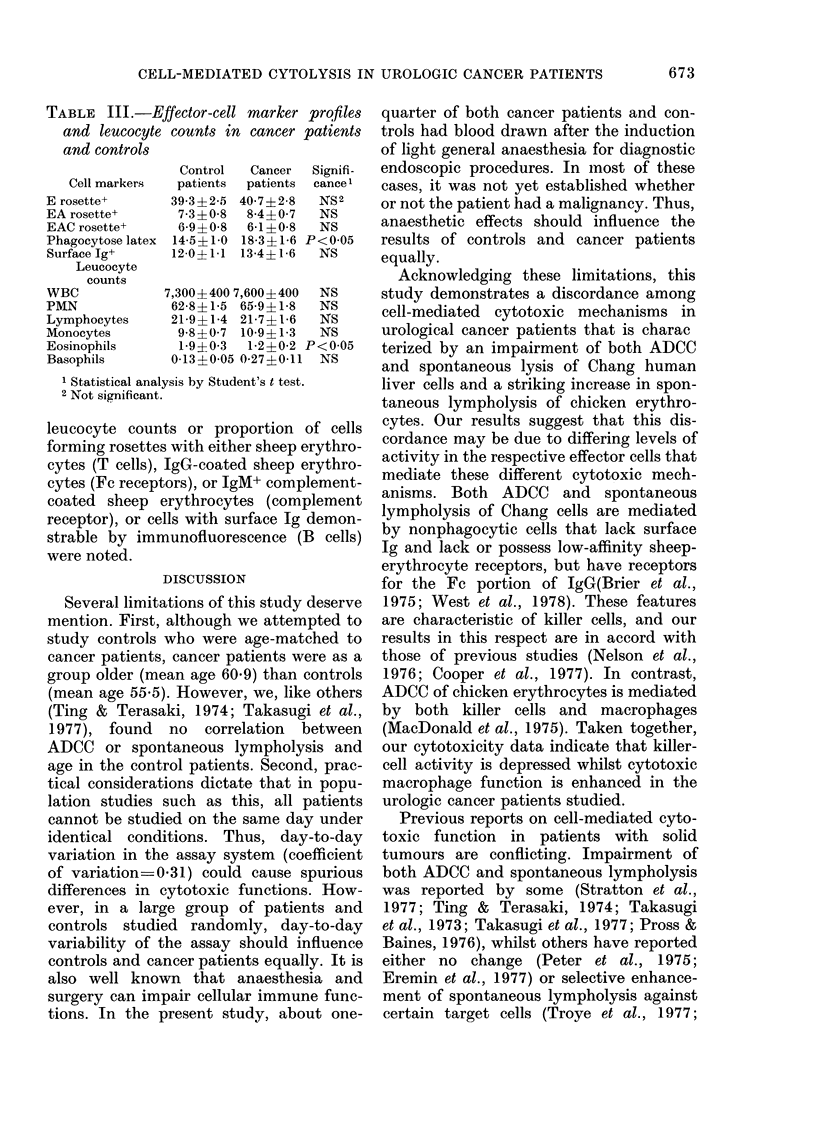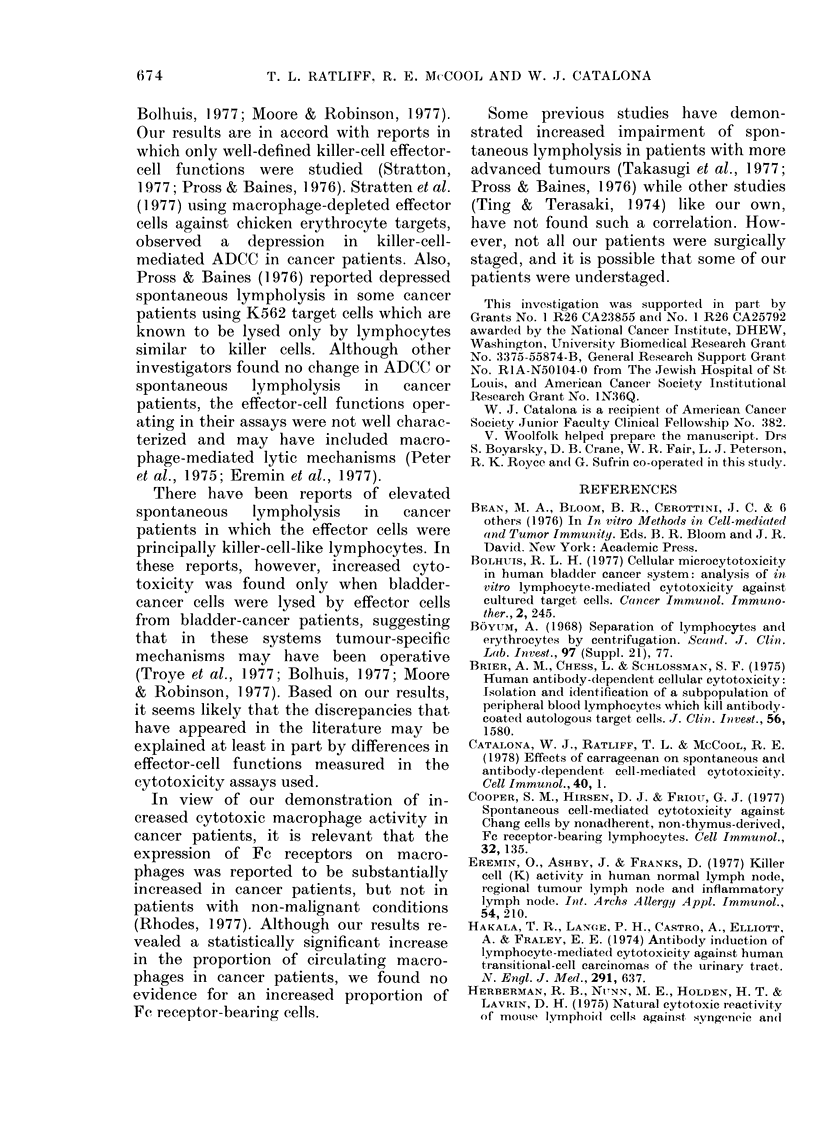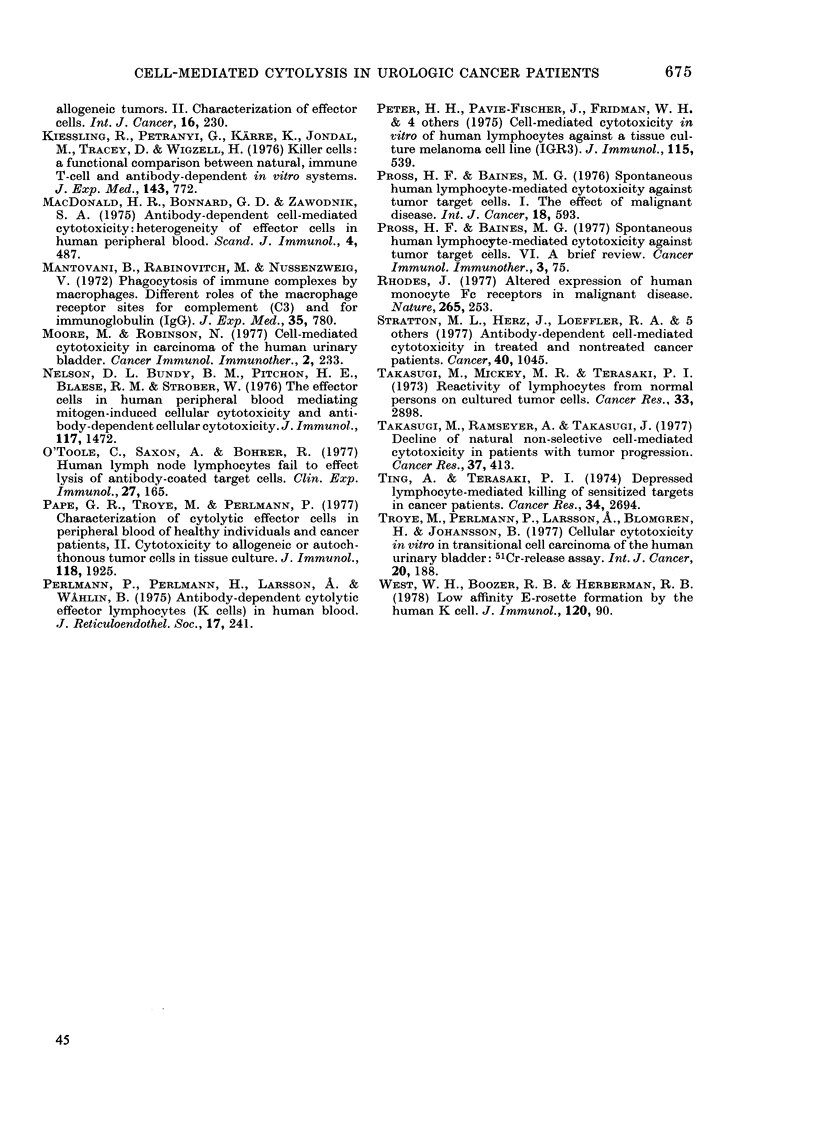# Antibody-dependent and spontaneous lympholysis in urologic cancer patients.

**DOI:** 10.1038/bjc.1979.118

**Published:** 1979-06

**Authors:** T. L. Ratliff, R. E. McCool, W. J. Catalona

## Abstract

To evaluate cytotoxic function mediated by killer lymphocytes and macrophages in urological cancer patients, we examined antibody-dependent and spontaneous lympholysis of chicken erythrocyte target cells, which is mediated by macrophages. Our results demonstrate a discordance between cytotoxic mechanisms in cancer patients, killer-cell function being impaired whilst macrophage-mediated cytotoxicity was increased.


*Br. J. Cancer* (1979) **39**, 667

### ANTIBODY-DEPENDENT AND SPONTANEOUS LYMPHOLYSIS IN UROLOGIC CANCER PATIENTS

T. L. RATLIFF,[1] R. E. McCOOL[1] AND W. J. CATALONA[1,2,3]

*From [1]The Yalem Research Laboratories of the Jewish Hospital of St Louis, and the [2]Department of Surgery (Urology), Washington University School of Medicine, St Louis, Missouri* 63110, *U.S.A.*

Received 3 January 1979 Accepted 12 February 1979

**Summary.—To evaluate cytotoxic function mediated by killer lymphocytes and macrophages in urological cancer patients, we examined antibody-dependent and spontaneous lympholysis of chicken erythrocyte target cells, which is mediated by macrophages. Our results demonstrate a discordance between cytotoxic mechanisms in cancer patients, killer-cell function being impaired whilst macrophage-mediated cytotoxicity was increased.**

RECENT STUDIES show that there are subpopulations of human lymphoid cells that are cytotoxic *in vitro* without prior sensitization to various target cells, including tumour cells (Perlmann *et al.*, 1975; Pross & Baines, 1977). Cytotoxicity by unsensitized lymphoid cells is greatly enhanced when target cells are coated with specific antibody (Perlmann *et al.*, 1975). This cytotoxic phenomenon is called antibody-dependent cell-mediated cytotoxicity or antibody-dependent lympholysis (ADCC). Cells that mediate ADCC have been operationally defined as killer cells (Perlmann *et al.*, 1975; Brier *et al.*, 1975).

It has been demonstrated that various types of effector cells can mediate ADCC depending upon the type of target cell. For example, antibody-coated chicken erythrocyte targets are susceptible to lysis by granulocytes, macrophages, and killer cells (MacDonald *et al.*, 1975) whilst antibody-coated Chang human liver cells (Nelson *et al.*, 1976) and many tumour-cell lines (O'Toole *et al.*, 1977) are susceptible to lysis only by killer cells.

Unsensitized lymphocytes are also capable of lysing target cells that are not coated with antibody, a process which will be referred to here as spontaneous lympholysis (Pross & Baines, 1976). As with ADCC, different types of effector cells are active in mediating spontaneous lympholysis, depending on the type of target cell. For example, macrophages can effect spontaneous lympholysis against chicken erythrocytes (Mantovani *et al.*, 1972) whereas in humans lymphocytes with surface properties similar to killer cells mediate spontaneous lympholysis against Chang cells (Cooper *et al.*, 1977) and many tumour cells (Pape *et al.*, 1977).

Little is known about the *in vivo* relevance of ADCC or spontaneous lympholysis in humans. ADCC has been shown by Hakala *et al.* (1974) to be operative in some patients with transitional-cell carcinoma, and therefore may be of clinical importance. Moreover, in animal tumour systems, spontaneous lympholysis has been postulated a natural cell-mediated surveillance mechanism against oncogenic viruses and tumour cells (Kiessling *et al.*, 1976; Herberman *et al.*, 1975) and a similar role has been postulated in humans (Pross & Baines, 1977).

In the present study we have examined simultaneously both ADCC and spon-

[3] Requests for reprints: 4960 Audubon Avenue, St Louis, Missouri 63110.

taneous lympholysis against two target-cell lines that are susceptible to lysis by different types of effector cells in 41 patients with genitourinary cancer and 59 age-matched controls with benign urological conditions. Our results demonstrate a discordance between cytotoxic mechanisms in cancer patients. Killer-cell-mediated lysis and non-macrophage-mediated spontaneous lympholysis were diminished, and macrophage-mediated cytotoxic mechanisms were markedly enhanced in comparison with controls.

### PATIENTS

Of the 100 patients studied, 41 were cancer patients (mean age 60·9) and 59 were controls with benign urological conditions (mean age 55·5). Six cancer patients and 5 controls were females. Patients receiving radiation or chemotherapy are not included in this report. Blood was drawn before major surgery in all cases. In about one quarter of both cancer patients and controls, blood was drawn immediately after the induction of anaesthesia for diagnostic endoscopic procedures. In many instances it was unknown whether patients were controls or cancer patients until after testing. Also, in many instances both cancer patients and controls were tested simultaneously. Thus, practical considerations required that both cancer patients and controls be studied randomly during the period of this investigation. The histological tumour types of the cancer patients are shown in Table I.

TABLE I.—*Cancer patients and controls tested for ADCC and spontaneous and antibody-dependent lympholysis*

| Patient group | No. of patients | Mean age ±s.e. |
|---|---|---|
| Control (benign urological conditions) | 59 | 55·5±2·2 |
| Bladder carcinoma | 13 | 62·7±3·6 |
| Prostate carcinoma | 18 | 66·1±1·4 |
| Testis tumours | 4 | 34·3±3·0 |
| Renal carcinoma | 3 | 61·3±5·4 |
| Miscellaneous[1] | 3 | 60·3±5·3 |
| Total cancer | 41 | 60·9±1·5 |

[1] 2 colon carcinoma, 1 uterine carcinoma.

### MATERIALS AND METHODS

We have previously reported most of the methods used in this study (Catalona *et al.*, 1978). These methods will be referred to here only briefly or by reference.

*Effector cells.*—Peripheral blood was drawn into a syringe containing 500 u of preservative-free heparin and was diluted with an equal volume of Hanks' balanced salt solution. Mononuclear cells were separated on a Ficoll–Hypaque density gradient as described by Böyum (1968) and were washed ×3 with tissue-culture medium. This procedure yielded 99% viable mononuclear cells with less than 2% granulocyte contamination.

*Target cells.*—Two types of target cell were used, chicken erythrocytes and Chang human liver cells, both of which have been well characterized for both spontaneous and antibody-dependent lympholysis (MacDonald *et al.*, 1975; Nelson *et al.*, 1976; Mantovani *et al.*, 1972; Cooper *et al.*, 1977). Target cells were labelled by incubating with 100 μCi or radioactive sodium dichromate for 60 min. Labelled target cells were washed and resuspended in tissue-culture medium (Catalona *et al.*, 1978).

*ADCC and spontaneous lympholysis assays.*—Chromium-labelled target cells were pipetted into triplicate wells of microtitre plates and incubated for 1 h with an appropriate dilution of an anti-target-cell serum. Serial dilutions of effector lymphocytes were then added to the antibody-coated target cells to give effector:target cell ratios of 50:1, 25:1, 12:1, 6:1, 3:1 for Chang cells and 5:1, 2·5:1, 6:1, 3:1 for chicken erythrocytes. Control cultures were set up using targets that were preincubated with normal rabbit serum instead of anti-target-cell serum. Additional controls included target cells alone (spontaneous release), target cells plus 4% cetrimide, a lysing detergent (maximal release), target cells plus anti-target serum, and target cells plus normal rabbit serum (Catalona *et al.*, 1978).

The microtitre plates were centrifuged and then incubated in a 37°C, 5% $CO_2$ incubator for 18 h for chicken erythrocytes and 4 h for Chang cells. The isotope released from target cells into the supernatant was quantitated in a gamma counter.

The following standard formulae (Cooper *et al.*, 1977) were used to calculate cytotoxicity:

$$\% \text{ ADCC} = \frac{\text{experimental release} - \text{release by highest control}}{\text{maximal release} - \text{release by highest control}} \times 100$$

$$\% \text{ spontaneous} = \frac{\text{release with lymphocytes and normal rabbit serum} - \text{release with normal rabbit serum only}}{\text{maximal release} - \text{release with normal rabbit serum only}} \times 100$$

The coefficient of variation for replicate samples from the same patient tested on the same day (within-assay variability) was 0·14, and the coefficient of variation for assays performed on the same patient on different days (between-assay variability) was 0·31.

*Effector-cell fractionation.*—To determine which cells mediated ADCC and spontaneous lysis of our target cells, peripheral-blood mononuclear cells were fractionated (courtesy of R. P. McDermott) by a modification of the technique described by Brier *et al.* (1975). Briefly, monocytes were removed by passage over a Sephadex G10 column or by glass adherence and subsequently removed. Monocyte-depleted lymphocytes were incubated at 37°C for 1 h to remove nonspecifically absorbed immunoglobulin, and were then passed over a Sephadex G-200 anti-human $(Fab')_2$ immunoadsorbent column. The T-cell-enriched population thus obtained was fractionated further by overnight sheep-erythrocyte rosette formation, followed by Ficoll–Hypaque density sedimentation. Non-sheep-erythrocyte-rosette-forming cells (null cells) at the interface were collected. T cells in the B-cell population were removed by overnight sheep-erythrocyte rosette formation at 4°C followed by Ficoll–Hypaque sedimentation. Fc receptor$^+$ cells were isolated by rosette formation with IgG-coated sheep erythrocytes at 37°C for 4 h followed by Ficoll–Hypaque sedimentation. Interface cells were collected as Fc receptor$^-$ cells.

*Effector-cell marker profiles* (Bean *et al.*, 1976).—We evaluated patient blood samples for total and differential leucocyte counts. We also evaluated the respective proportions of mononuclear cells forming spontaneous rosettes with sheep erythrocytes (T cells), cells with demonstrable surface immunoglobulin (B cells), cells forming rosettes with IgG-coated sheep erythrocytes that had been

TABLE II.—*ADCC and spontaneous lympholysis by peripheral-blood mononuclear cell subpopulations from a normal volunteer against chicken erythrocyte and Chang cells*

| | % ADCC lympholysis | | % Spontaneous lympholysis | |
|---|---|---|---|---|
| | Chang[1] | Chicken Eythrocyte[2] | Chang[3] | Chicken Erythrocyte |
| Unfractionated mononuclear cells | 37 | 49 | 10 | ND[5] |
| Macrophage-depleted mononuclear cells | 33 | 16 | 5 | ND |
| T+null | 33 | 9 | 2 | ND |
| T ($E^+$, $Fc^+$) | 30 | 6 | 23[6] | ND |
| T ($E^+$, $Fc^-$) | 15 | 0 | | ND |
| Surface $Ig^+$ | 2 | 0 | 3 | ND |
| Null | 66 | 35 | 35 | ND |
| Macrophage-enriched | 18 | 27 | 0 | ND |
| Unfractionated mononuclear cells[4] | ND | 28 | ND | 40 |
| Glass-nonadherent[4] | ND | 46 | ND | 2 |
| Glass-adherent[4] | ND | 20 | ND | 48 |

[1] $1{\cdot}2 \times 10^5$ effector cells per $5 \times 10^3$ targets with a 4h incubation.
[2] $2{\cdot}5 \times 10^6$ effector cells per $10^5$ targets with a 4h incubation. The anti-chicken erythrocyte dilution used was $10^{-3}$.
[3] $5 \times 10^5$ effector cells per $10^4$ targets with a 36h incubation.
[4] $2{\cdot}5 \times 10^5$ effector cells per $10^5$ targets with an 18h incubation.
[5] Not done.
[6] T ($E^+$, $Fc^{\pm}$).

previously coated with IgM antibody plus complement (complement-receptor-bearing lymphocytes) and cells phagocytosing latex particles (macrophages).

*Data analysis.*—We plotted curves depicting % cytotoxicity as a function of effector-cell concentration. Differences in cytotoxicity between patient groups were evaluated for significance by Student's $t$ test. We evaluated correlations between assays by regression analysis.

### RESULTS

### *Effector cells mediating ADCC and spontaneous lympholysis*

Fractionation profiles were performed on cells obtained from 3 normal volunteers on separate days. Although the absolute lytic activity varied from donor to donor, the relative lytic activity in each of the fractions was comparable in all experiments. A representative experiment is shown in Table II. The results showed that ADCC of Chang cells was mediated primarily by nonphagocytic cells that lacked surface immunoglobulin and many of which lacked receptors for sheep erythrocytes, but expressed Fc receptors. These data are consistent with previous reports (Nelson *et al.*, 1976) on killer-cell activity against Chang cells. ADCC of chicken erythrocytes was mediated by both killer cells and macrophages, which is also in accord with previous reports (MacDonald *et al.*, 1975).

FIG. 1.—ADCC of Chang cells in patients and controls.

FIG. 2.—ADCC of Chang cells in patients with bladder or prostate carcinoma and controls.

FIG. 3.—Effect of tumour stage on ADCC of Chang cells.

FIG. 4.—Spontaneous lympholysis of Chang cells in cancer patients and controls.

FIG. 5.—Spontaneous lympholysis of Chang cells in patients with bladder or prostate carcinoma and controls.

Spontaneous lympholysis of Chang cells (Table II) was mediated by nonphagocytic cells that lacked immunoglobulin, but some of which had receptors for sheep erythrocytes (West *et al.*, 1978). On the other hand, spontaneous lympholysis of chicken erythrocytes was mediated primarily by macrophages (Table II) (Mantovani *et al.*, 1972).

*Relationship between ADCC and spontaneous lympholysis and age*

Regression analysis revealed no correlation between either ADCC or spontaneous lympholysis and patient age (data not shown).

*ADCC and spontaneous lympholysis of Chang cells*

ADCC of Chang cells was depressed in cancer patients (Fig. 1). This depression was statistically significant ($P<0{\cdot}05$) at 25:1 and 50:1 effector : target cell ratios and in terms of peak cytotoxicity. Both bladder and prostate-cancer patients had peak lytic levels that were significantly less than the controls (Fig. 2). Interestingly, patients with clinically localized tumours showed a greater impairment of ADCC of Chang cells than patients with metastases (Fig. 3). However, not all patients studied were surgically staged.

Spontaneous lympholysis of Chang cells was also significantly depressed in cancer patients (Fig. 4). Both prostate and bladder-cancer patients had significant impairments of spontaneous lysis at some effector:target cell ratios; however, the impairment was more pronounced in prostate-cancer patients (Fig. 5). There was no correlation between the impair-

FIG. 6.—ADCC of chicken erythrocytes target cells in cancer patients and controls.

ment of spontaneous lympholysis and the extent of the tumour involvement.

*ADCC and spontaneous lympholysis of chicken erythrocytes*

We found no significant difference in ADCC of chicken erythrocytes between cancer patients and controls (Fig. 6). Bladder-cancer patients showed stronger lysis than either prostate-cancer patients or controls, but the difference was not statistically significant (Fig. 7). There was also no correlation between ADCC of chicken erythrocytes and tumour stage.

In contrast, we observed a highly significant increase in spontaneous lympholysis of chicken erythrocytes in cancer patients (Fig. 8). This increase was significant for both prostate and bladder-cancer patients (Fig. 9), but there was no correlation between tumour stage and spontaneous lysis of chicken erythrocytes.

*Leucocyte counts and effector-cell marker profiles*

Cancer patients had a significantly higher proportion of latex-ingesting mononuclear cells (macrophages) and lower proportion of eosinophils than controls (Table III). No significant differences in

FIG. 8.—Spontaneous lympholysis of chicken erythrocytes in cancer patients and controls.

FIG. 7.—ADCC of chicken erythrocytes cells in bladder or prostate carcinoma patients and controls.

FIG. 9.—Spontaneous lympholysis of chicken erythrocytes in bladder or prostate-carcinoma patients and controls.

TABLE III.—*Effector-cell marker profiles and leucocyte counts in cancer patients and controls*

| Cell markers | Control patients | Cancer patients | Significance[1] |
|---|---|---|---|
| E rosette$^+$ | 39·3±2·5 | 40·7±2·8 | NS[2] |
| EA rosette$^+$ | 7·3±0·8 | 8·4±0·7 | NS |
| EAC rosette$^+$ | 6·9±0·8 | 6·1±0·8 | NS |
| Phagocytose latex | 14·5±1·0 | 18·3±1·6 | $P<0·05$ |
| Surface Ig$^+$ | 12·0±1·1 | 13·4±1·6 | NS |
| Leucocyte counts | | | |
| WBC | 7,300±400 | 7,600±400 | NS |
| PMN | 62·8±1·5 | 65·9±1·8 | NS |
| Lymphocytes | 21·9±1·4 | 21·7±1·6 | NS |
| Monocytes | 9·8±0·7 | 10·9±1·3 | NS |
| Eosinophils | 1·9±0·3 | 1·2±0·2 | $P<0·05$ |
| Basophils | 0·13±0·05 | 0·27±0·11 | NS |

[1] Statistical analysis by Student's *t* test.
[2] Not significant.

leucocyte counts or proportion of cells forming rosettes with either sheep erythrocytes (T cells), IgG-coated sheep erythrocytes (Fc receptors), or IgM$^+$ complement-coated sheep erythrocytes (complement receptor), or cells with surface Ig demonstrable by immunofluorescence (B cells) were noted.

### DISCUSSION

Several limitations of this study deserve mention. First, although we attempted to study controls who were age-matched to cancer patients, cancer patients were as a group older (mean age 60·9) than controls (mean age 55·5). However, we, like others (Ting & Terasaki, 1974; Takasugi *et al.*, 1977), found no correlation between ADCC or spontaneous lympholysis and age in the control patients. Second, practical considerations dictate that in population studies such as this, all patients cannot be studied on the same day under identical conditions. Thus, day-to-day variation in the assay system (coefficient of variation=0·31) could cause spurious differences in cytotoxic functions. However, in a large group of patients and controls studied randomly, day-to-day variability of the assay should influence controls and cancer patients equally. It is also well known that anaesthesia and surgery can impair cellular immune functions. In the present study, about one-quarter of both cancer patients and controls had blood drawn after the induction of light general anaesthesia for diagnostic endoscopic procedures. In most of these cases, it was not yet established whether or not the patient had a malignancy. Thus, anaesthetic effects should influence the results of controls and cancer patients equally.

Acknowledging these limitations, this study demonstrates a discordance among cell-mediated cytotoxic mechanisms in urological cancer patients that is characterized by an impairment of both ADCC and spontaneous lysis of Chang human liver cells and a striking increase in spontaneous lympholysis of chicken erythrocytes. Our results suggest that this discordance may be due to differing levels of activity in the respective effector cells that mediate these different cytotoxic mechanisms. Both ADCC and spontaneous lympholysis of Chang cells are mediated by nonphagocytic cells that lack surface Ig and lack or possess low-affinity sheep-erythrocyte receptors, but have receptors for the Fc portion of IgG(Brier *et al.*, 1975; West *et al.*, 1978). These features are characteristic of killer cells, and our results in this respect are in accord with those of previous studies (Nelson *et al.*, 1976; Cooper *et al.*, 1977). In contrast, ADCC of chicken erythrocytes is mediated by both killer cells and macrophages (MacDonald *et al.*, 1975). Taken together, our cytotoxicity data indicate that killer-cell activity is depressed whilst cytotoxic macrophage function is enhanced in the urologic cancer patients studied.

Previous reports on cell-mediated cytotoxic function in patients with solid tumours are conflicting. Impairment of both ADCC and spontaneous lympholysis was reported by some (Stratton *et al.*, 1977; Ting & Terasaki, 1974; Takasugi *et al.*, 1973; Takasugi *et al.*, 1977; Pross & Baines, 1976), whilst others have reported either no change (Peter *et al.*, 1975; Eremin *et al.*, 1977) or selective enhancement of spontaneous lympholysis against certain target cells (Troye *et al.*, 1977;

Bolhuis, 1977; Moore & Robinson, 1977). Our results are in accord with reports in which only well-defined killer-cell effector-cell functions were studied (Stratton, 1977; Pross & Baines, 1976). Stratten *et al.* (1977) using macrophage-depleted effector cells against chicken erythrocyte targets, observed a depression in killer-cell-mediated ADCC in cancer patients. Also, Pross & Baines (1976) reported depressed spontaneous lympholysis in some cancer patients using K562 target cells which are known to be lysed only by lymphocytes similar to killer cells. Although other investigators found no change in ADCC or spontaneous lympholysis in cancer patients, the effector-cell functions operating in their assays were not well characterized and may have included macrophage-mediated lytic mechanisms (Peter *et al.*, 1975; Eremin *et al.*, 1977).

There have been reports of elevated spontaneous lympholysis in cancer patients in which the effector cells were principally killer-cell-like lymphocytes. In these reports, however, increased cytotoxicity was found only when bladder-cancer cells were lysed by effector cells from bladder-cancer patients, suggesting that in these systems tumour-specific mechanisms may have been operative (Troye *et al.*, 1977; Bolhuis, 1977; Moore & Robinson, 1977). Based on our results, it seems likely that the discrepancies that have appeared in the literature may be explained at least in part by differences in effector-cell functions measured in the cytotoxicity assays used.

In view of our demonstration of increased cytotoxic macrophage activity in cancer patients, it is relevant that the expression of Fc receptors on macrophages was reported to be substantially increased in cancer patients, but not in patients with non-malignant conditions (Rhodes, 1977). Although our results revealed a statistically significant increase in the proportion of circulating macrophages in cancer patients, we found no evidence for an increased proportion of Fc receptor-bearing cells.

Some previous studies have demonstrated increased impairment of spontaneous lympholysis in patients with more advanced tumours (Takasugi *et al.*, 1977; Pross & Baines, 1976) while other studies (Ting & Terasaki, 1974) like our own, have not found such a correlation. However, not all our patients were surgically staged, and it is possible that some of our patients were understaged.

This investigation was supported in part by Grants No. 1 R26 CA23855 and No. 1 R26 CA25792 awarded by the National Cancer Institute, DHEW, Washington, University Biomedical Research Grant No. 3375-55874-B, General Research Support Grant No. R1A-N50104-0 from The Jewish Hospital of St Louis, and American Cancer Society Institutional Research Grant No. IN36Q.

W. J. Catalona is a recipient of American Cancer Society Junior Faculty Clinical Fellowship No. 382.

V. Woolfolk helped prepare the manuscript. Drs S. Boyarsky, D. B. Crane, W. R. Fair, L. J. Peterson, R. K. Royce and G. Sufrin co-operated in this study.

### REFERENCES

Bean, M. A., Bloom, B. R., Cerottini, J. C. & 6 others (1976) In *In vitro Methods in Cell-mediated and Tumor Immunity*. Eds. B. R. Bloom and J. R. David. New York: Academic Press.

Bolhuis, R. L. H. (1977) Cellular microcytotoxicity in human bladder cancer system: analysis of *in vitro* lymphocyte-mediated cytotoxicity against cultured target cells. *Cancer Immunol. Immunother.*, **2**, 245.

Böyum, A. (1968) Separation of lymphocytes and erythrocytes by centrifugation. *Scand. J. Clin. Lab. Invest.*, **97** (Suppl. 21), 77.

Brier, A. M., Chess, L. & Schlossman, S. F. (1975) Human antibody-dependent cellular cytotoxicity: Isolation and identification of a subpopulation of peripheral blood lymphocytes which kill antibody-coated autologous target cells. *J. Clin. Invest.*, **56**, 1580.

Catalona, W. J., Ratliff, T. L. & McCool, R. E. (1978) Effects of carrageenan on spontaneous and antibody-dependent cell-mediated cytotoxicity. *Cell Immunol.*, **40**, 1.

Cooper, S. M., Hirsen, D. J. & Friou, G. J. (1977) Spontaneous cell-mediated cytotoxicity against Chang cells by nonadherent, non-thymus-derived, Fc receptor-bearing lymphocytes. *Cell Immunol.*, **32**, 135.

Eremin, O., Ashby, J. & Franks, D. (1977) Killer cell (K) activity in human normal lymph node, regional tumour lymph node and inflammatory lymph node. *Int. Archs Allergy Appl. Immunol.*, **54**, 210.

Hakala, T. R., Lange, P. H., Castro, A., Elliott, A. & Fraley, E. E. (1974) Antibody induction of lymphocyte-mediated cytotoxicity against human transitional-cell carcinomas of the urinary tract. *N. Engl. J. Med.*, **291**, 637.

Herberman, R. B., Nunn, M. E., Holden, H. T. & Lavrin, D. H. (1975) Natural cytotoxic reactivity of mouse lymphoid cells against syngeneic and allogeneic tumors. II. Characterization of effector cells. *Int. J. Cancer*, **16**, 230.

KIESSLING, R., PETRANYI, G., KÄRRE, K., JONDAL, M., TRACEY, D. & WIGZELL, H. (1976) Killer cells: a functional comparison between natural, immune T-cell and antibody-dependent *in vitro* systems. *J. Exp. Med.*, **143**, 772.

MACDONALD, H. R., BONNARD, G. D. & ZAWODNIK, S. A. (1975) Antibody-dependent cell-mediated cytotoxicity: heterogeneity of effector cells in human peripheral blood. *Scand. J. Immunol.*, **4**, 487.

MANTOVANI, B., RABINOVITCH, M. & NUSSENZWEIG, V. (1972) Phagocytosis of immune complexes by macrophages. Different roles of the macrophage receptor sites for complement (C3) and for immunoglobulin (IgG). *J. Exp. Med.*, **35**, 780.

MOORE, M. & ROBINSON, N. (1977) Cell-mediated cytotoxicity in carcinoma of the human urinary bladder. *Cancer Immunol. Immunother.*, **2**, 233.

NELSON, D. L. BUNDY, B. M., PITCHON, H. E., BLAESE, R. M. & STROBER, W. (1976) The effector cells in human peripheral blood mediating mitogen-induced cellular cytotoxicity and antibody-dependent cellular cytotoxicity. *J. Immunol.*, **117**, 1472.

O'TOOLE, C., SAXON, A. & BOHRER, R. (1977) Human lymph node lymphocytes fail to effect lysis of antibody-coated target cells. *Clin. Exp. Immunol.*, **27**, 165.

PAPE, G. R., TROYE, M. & PERLMANN, P. (1977) Characterization of cytolytic effector cells in peripheral blood of healthy individuals and cancer patients, II. Cytotoxicity to allogeneic or autochthonous tumor cells in tissue culture. *J. Immunol.*, **118**, 1925.

PERLMANN, P., PERLMANN, H., LARSSON, Å. & WÅHLIN, B. (1975) Antibody-dependent cytolytic effector lymphocytes (K cells) in human blood. *J. Reticuloendothel. Soc.*, **17**, 241.

PETER, H. H., PAVIE-FISCHER, J., FRIDMAN, W. H. & 4 others (1975) Cell-mediated cytotoxicity *in vitro* of human lymphocytes against a tissue culture melanoma cell line (IGR3). *J. Immunol.*, **115**, 539.

PROSS, H. F. & BAINES, M. G. (1976) Spontaneous human lymphocyte-mediated cytotoxicity against tumor target cells. I. The effect of malignant disease. *Int. J. Cancer*, **18**, 593.

PROSS, H. F. & BAINES, M. G. (1977) Spontaneous human lymphocyte-mediated cytotoxicity against tumor target cells. VI. A brief review. *Cancer Immunol. Immunother.*, **3**, 75.

RHODES, J. (1977) Altered expression of human monocyte Fc receptors in malignant disease. *Nature*, **265**, 253.

STRATTON, M. L., HERZ, J., LOEFFLER, R. A. & 5 others (1977) Antibody-dependent cell-mediated cytotoxicity in treated and nontreated cancer patients. *Cancer*, **40**, 1045.

TAKASUGI, M., MICKEY, M. R. & TERASAKI, P. I. (1973) Reactivity of lymphocytes from normal persons on cultured tumor cells. *Cancer Res.*, **33**, 2898.

TAKASUGI, M., RAMSEYER, A. & TAKASUGI, J. (1977) Decline of natural non-selective cell-mediated cytotoxicity in patients with tumor progression. *Cancer Res.*, **37**, 413.

TING, A. & TERASAKI, P. I. (1974) Depressed lymphocyte-mediated killing of sensitized targets in cancer patients. *Cancer Res.*, **34**, 2694.

TROYE, M., PERLMANN, P., LARSSON, Å., BLOMGREN, H. & JOHANSSON, B. (1977) Cellular cytotoxicity *in vitro* in transitional cell carcinoma of the human urinary bladder: $^{51}$Cr-release assay. *Int. J. Cancer*, **20**, 188.

WEST, W. H., BOOZER, R. B. & HERBERMAN, R. B. (1978) Low affinity E-rosette formation by the human K cell. *J. Immunol.*, **120**, 90.